# The Ship's Doctor

**Published:** 1912-02-24

**Authors:** 


### THE SHIP'S DOCTOR.

Members of the profession who "go down to the sea in ships" have hitherto had the privilege of constituting a class by themselves—a class which it has been difficult to regard without a feeling of mingled envy and pity. Envy because the ship's doctor gets the opportunity, which is denied to his stay-at-home colleague, of seeing the world, of travelling, usually under excellent conditions, to whatever part of the universe he pleases—since his choice of destination is only limited by the number of English ships that require a surgeon—and of making friends with all sorts and conditions of men. Pity because hitherto there has been an uncomfortable suspicion, amounting almost to a certainty, that he was being underpaid, or sweated as the popular term is. True, against this abnormally low rate of pay—which works out on an average at a guinea a week—must be set the compensatory advantages already enumerated, and to the sum on the credit side must be added the fact that in many cases the work demanded from him is of an extremely elementary nature, so much so that in some cases his post is, in fact, a sinecure. That, however, does not alter the equally indubitable fact that he has a position of responsibility, and that he devotes a large amount of time to the service of the company which employs him.

In these circumstances it is satisfactory to note that there is every possibility that the ship's surgeon is at last getting that justice of treatment which he has vainly asked for during the past century. Some of the great passenger-carrying lines have now adopted the principle of allowing the surgeon in charge of a ship to demand payment for his services from first-class passengers. When it is remembered that a large steamship carries hundreds of passengers, every one of whom has the right—or has hitherto had the right—of demanding free treatment and advice from the ship's doctor, it will easily be understood how revolutionary the new régime promises to be. In the past the ship's doctor has had to work his hardest on board these ships, although his rate of payment was no greater than that granted to surgeons staffing smaller boats where the service was much less onerous. The greater part of such work has been done gratuitously. Where a passenger belonging to the rare genus of the grateful patient so cordially appreciated the care and attention paid to him on the voyage as to testify his gratitude in a practical shape, that testimony has always been in the nature of a "tip"—a form of remuneration common enough, as the butler in one of Mr. Shaw's plays remarks, but one which is particularly unsuitable for medical work. It is therefore not to be wondered that the impression has arisen that the ship's doctor, as a class, does inferior work as compared with his colleague the private practitioner on land. Nothing can be further from the truth than this supposition. Ships' doctors, as a class, are very able, careful, and experienced men; as practitioners they compare very favourably with their colleagues who stay at home. Nor can it be honestly asserted that they give less attention to first-class patients than to any others under their charge, or work more conscientiously in anticipation of a tip than in cases where they know that such a bounty is quite out of the question. In addition they are exposed to the same danger, arising out of an action for malpraxis, as their colleagues. That being the case, it is only right that they should be allowed to look upon the mass of paying passengers as private patients. The small official remuneration they receive is in the nature of a retaining fee for work to be done in the interests of the crew. We should like the principle now recognised, so far as the first-class passengers are concerned, extended so as to include all paying passengers on board, the fees to be charged to be arranged on a scale in accordance with that obtaining at the port from which the ship has sailed. At present the fees charged are subject to the approval of the captain, a proviso which should be deleted, since in case of overcharge an appeal to the captain is the first step that an irate passenger is likely to take. A beginning has been made; to follow it up is the work of all ships' surgeons who are cognisant of the value of co-operation and combination.

